# Tris(acetonitrile-κ*N*){2,6-bis­[(diphenyl­phosphan­yl)amino]-4-eth­oxy-1,3,5-triazine-κ^3^ *P*,*N* ^1^,*P*′}iron(II) bis­(tetra­fluorido­borate) acetonitrile disolvate

**DOI:** 10.1107/S1600536811049804

**Published:** 2011-11-25

**Authors:** Moumita Koley, Karl Kirchner, Kurt Mereiter

**Affiliations:** a Institute of Applied Synthetic Chemistry, Vienna University of Technology, Getreidemarkt 9/163, A-1060 Vienna, Austria; b Institute of Chemical Technology and Analytics, Vienna University of Technology, Getreidemarkt 9/164SC, A-1060 Vienna, Austria

## Abstract

In the title compound, [Fe(CH_3_CN)_3_(C_29_H_27_N_5_OP_2_)](BF_4_)_2_·2CH_3_CN, the Fe^II^ ion is octa­hedrally coordinated by a meridionally chelating tridentate pincer-type PNP ligand derived from 2,6-diamino-4-eth­oxy-1,3,5-triazine and by three acetonitrile mol­ecules. The four Fe—N bond lengths range from 1.9142 (12) to 1.9579 (11) Å, while the Fe—P bonds are 2.2452 (4) and 2.2506 (4) Å [P—Fe—P = 165.523 (14)°], consistent with Fe^II^ in a low-spin state. Unlike related Fe PNP complexes based on 2,6-diamino­pyridine, the BF_4_ anions are not hydrogen bonded to the two NH groups of the pincer ligand but show instead anion–π inter­actions with the triazine ring and acetonitrile mol­ecules in addition to ten C—H⋯F inter­actions. Most remarkable among these is an anion–π(triazine) inter­action with a short distance of 2.788 (2) Å between one F and the centroid of the π-acidic triazine ring. The corresponding shortest distance between this F atom and a triazine carbon atom is 2.750 (2) Å. The two NH groups of the pincer ligand donate N—H⋯N hydrogen bonds to the triazine N atom of a neighbouring complex and to an uncoordinated acetonitrile mol­ecule. This last mol­ecule is in a side-on head-to-tail association with the second uncoordinated acetonitrile at C⋯N distances of 3.467 (2) and 3.569 (2) Å. In contrast to several related compounds with diamino­pyridine- instead of diamino­triazine-based PNP ligands, the title crystal structure is remarkably well ordered. This suggests that the diamino­triazine moiety exerts notable crystal structure stabilizing effects.

## Related literature

For a review on PNP and PCP pincer complexes based on 2,6-diamino­pyridine and 1,3-diamino­benzene, see: Benito-Garagorri & Kirchner (2008 ▶). For the crystal structures of related PNP pincer complexes, see: Benito-Garagorri *et al.* (2006 ▶). For weak hydrogen bonds, see Desiraju & Steiner (1999 ▶). For anion–π inter­actions, see Gamez *et al.* (2007 ▶); Mooibroek *et al.* (2008 ▶); Manzano *et al.* (2008 ▶); Quinonero *et al.* (2010 ▶); Lu *et al.* (2009 ▶). For a description of the Cambridge Structural Database, see: Allen (2002 ▶).
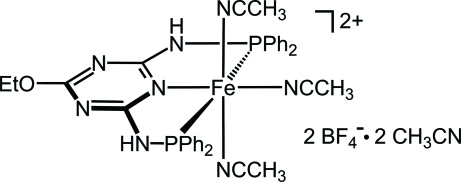


## Experimental

### 

#### Crystal data

[Fe(C_2_H_3_N)_3_(C_29_H_27_N_5_OP_2_)](BF_4_)_2_·2C_2_H_3_N
*M* *_r_* = 958.24
Triclinic, 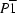

*a* = 8.8548 (4) Å
*b* = 13.8402 (7) Å
*c* = 20.1352 (10) Å
α = 71.399 (2)°
β = 82.731 (2)°
γ = 72.789 (2)°
*V* = 2232.6 (2) Å^3^
*Z* = 2
Mo *K*α radiation
μ = 0.49 mm^−1^
*T* = 100 K
0.58 × 0.36 × 0.30 mm

#### Data collection

Bruker SMART APEX CCD diffractometer
Absorption correction: multi-scan (*SADABS*; Bruker, 2003 ▶) *T*_min_ = 0.74, *T*_max_ = 0.86
26262 measured reflections
12688 independent reflections
11076 reflections with *I* > 2σ(*I*)
*R*_int_ = 0.017

#### Refinement

*R*[*F* ^2^ > 2σ(*F* ^2^)] = 0.038
*wR*(*F* ^2^) = 0.102
*S* = 1.03
12688 reflections
573 parameters
H-atom parameters constrained
Δρ_max_ = 0.74 e Å^−3^
Δρ_min_ = −0.44 e Å^−3^

### 

Data collection: *SMART* (Bruker, 2003 ▶); cell refinement: *SAINT* (Bruker, 2003 ▶); data reduction: *SAINT*; program(s) used to solve structure: *SHELXS97* (Sheldrick, 2008 ▶); program(s) used to refine structure: *SHELXL97* (Sheldrick, 2008 ▶); molecular graphics: *SHELXTL* (Sheldrick, 2008 ▶) and *Mercury* (Macrae *et al.*, 2006 ▶); software used to prepare material for publication: *PLATON* (Spek, 2009 ▶) and *publCIF* (Westrip, 2010 ▶).

## Supplementary Material

Crystal structure: contains datablock(s) I, global. DOI: 10.1107/S1600536811049804/gk2437sup1.cif

Structure factors: contains datablock(s) I. DOI: 10.1107/S1600536811049804/gk2437Isup2.hkl

Additional supplementary materials:  crystallographic information; 3D view; checkCIF report

## Figures and Tables

**Table 1:** Hydrogen-bond geometry (Å, °)

*D*—H⋯*A*	*D*—H	H⋯*A*	*D*⋯*A*	*D*—H⋯*A*
N4—H4*N*⋯N9	0.88	2.03	2.892 (2)	168
N5—H5*N*⋯N3^i^	0.88	2.12	2.966 (2)	162
C9—H9⋯F7	0.95	2.53	3.276 (2)	135
C12—H12⋯F4	0.95	2.56	3.280 (2)	133
C13—H13⋯F2^ii^	0.95	2.54	3.464 (2)	164
C21—H21⋯F6	0.95	2.49	3.350 (2)	150
C25—H25⋯F5^iii^	0.95	2.42	3.365 (2)	174
C26—H26⋯F5^i^	0.95	2.50	3.144 (2)	125
C29—H29*A*⋯F3^iv^	0.98	2.35	3.239 (2)	151
C31—H31*A*⋯F8	0.98	2.46	3.360 (2)	153
C33—H33*B*⋯F4	0.98	2.49	3.191 (2)	128
C39—H39*B*⋯F2^v^	0.98	2.41	3.259 (3)	144

Symmetry codes: (i) 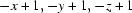; (ii) 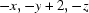; (iii) 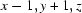; (iv) 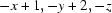; (v) 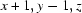.

**Fig. 1.**
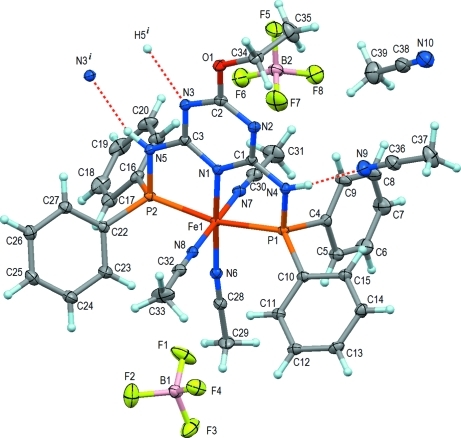
The asymmetric unit of (I) with displacement ellipsoids at the 50% probability level showing only N—H···N hydrogen bonds as red dashed lines. Symmetry code (i): 1 - x, 1 - y, 1 - z.

**Fig. 2.**
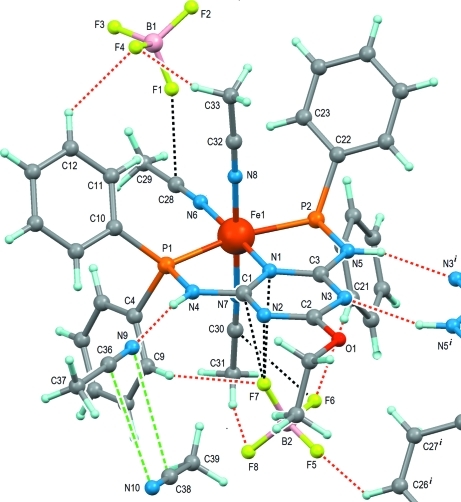
Selected hydrogen bonds, anion···π interactions, and head-to-tail association two CH3CN in (I) indicated by red, black, and green dashed lines, respectively. Cg is the centroid of the triazine ring. For geometric data of the hydrogen bonds, see Table 2. Other distances (Å) are: F1···C28 3.006 (2), F6···C30 3.091 (2), F7···C1 2.750 (2), F7···N2 2.915 (2), F7···N1 3.012 (2), N9···C38 3.569 (2), C36···N10 3.467 (2). Symmetry code (i): 1 - x, 1 - y, 1 - z.

**Fig. 3.**
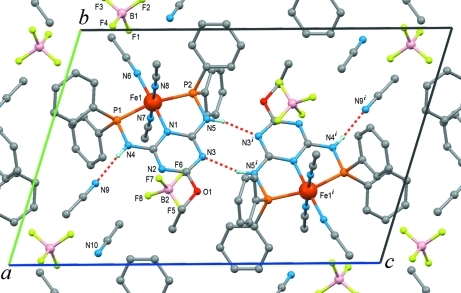
Crystal packing of (I) in a view along the a-axis showing how the N—H···N hydrogen bonds link two Fe complexes and two acetontrile solvate molecules into a finite group. Symmetry code (i): 1 - x, 1 - y, 1 - z.

## supplementary crystallographic information

### Comment

The title compound belongs to a family of transition metal complexes with tridentate PNP pincer ligands in which a central pyridine ring contains two –NH–P*R*_2_ substituents in the two *ortho*-positions (*R* = alkyl, aryl, alkoxy, aryloxy). Together with the pyridine N atom the two P atoms of the ligand chelate transition metals in meridional geometry and generate thereby robust complexes with interesting properties for applications like homogeneous catalysis (Benito-Garagorri & Kirchner, 2008). Instead of pyridine the title compound is based on 1,3,5-triazine substituted in the 2- and 6-positions by diphenylphosphinoamine groups and in the 4-position by an ethoxy group. Such modifications aim to alter the electronic properties of the PNP ligand and the solubility properties of the resulting complex.

In the title compound, (I), iron is in a distorted octahedral coordination by the PNP ligand and three acetonitrile molecules providing a P_2_N_4_ set of donor atoms (Fig. 1) with bond lengths to Fe1 listed in Table 1 and following further features: mean bond length Fe1—N = 1.931 (17) Å, mean bond length Fe1—P = 2.248 (4) Å, *cis* bond angles from 82.74 (3) to 99.61 (3)°, *trans* bond angles N1—Fe1—N6 = 177.64 (4)°, N7—Fe1—N8 = 179.78 (5)°, and P1—Fe1—P2 = 165.52 (2)°. These geometric features are similar to those of four related iron(II) PNP tris(acetonitrile) complexes that are based on pyridine and were reported by Benito-Garagorri *et al.* (2006) [refcodes JEGQAO, JEGQES, JEGQIW, JEGQOC of the Cambridge Structural Database, Version 5.31, with Aug. 2010 updates; Allen, 2002]. The remaining parts of the Fe complex in (I) show normal dimensions. The triazine ring has a mean bond lengths of 1.341 (11) Å and the familiar trigonal distortion with C—N—C bond angles near 114° and N—C—N bond angles in the range 124.0 (1) - 126.9 (1)°. The ethoxy group adopts an angular conformation with torsion angle C2—O1—C34—C35 = 89.2 (2)°.

Whereas the above mentioned related Fe PNP compounds are all plagued by orientation disorder of their BF_4_^-^ counter anions, the title compound (I) is well ordered with respect to BF_4_ and the two noncoordinating acetonitrile solvent molecules as well. This is interesting because the BF_4_ anions normally form N—H···F hydrogen bonds with the distinctly acidic NH groups of the PNP ligands (Benito-Garagorri *et al.*, 2006), while in (I) they do not. As outlined in Fig. 2, the N5—H group in (I) forms a pair of complementary N5—H···N3 hydrogen bonds with a centrosymmetric equivalent of the Fe complex (N5···N3 = 2.966 Å, Table 2), and the N4—H group a hydrogen bond to N9 of a noncoordinating acetonitrile molecule (N4···N9 = 2.892 Å). As indicated by the green dashed lines in Fig. 2, this CH_3_CN molecule is in close head-to-tail association with the second noncoordinating CH_3_CN molecule, N9···C38 = 3.569 (2) Å, C36···N10 = 3.467 (2) Å, helping to fix that molecule in the crystal lattice. The two BF_4_ anions in turn are engaged in C—H···F and anion–π interactions. There are ten C—H···F interactions with C···F distances in the range 3.144 (2) to 3.464 (2) Å (*cf.* Table 2) of which a part is shown in Fig. 2. These bonds are with six phenyl and four CH_3_CN hydrogen atoms (Desiraju & Steiner, 1999). Very interesting in (I) is the strong anion–π interaction between the B2F_4_ anion and the triazine ring, which is a distinctly π–acidic or electron deficient heteroaromat very suitable for such interaction: The distance between F7 and the triazine ring centroid *Cg*1 (*x*,*y*,*z* = 0.453645 0.475396 0.361507) is *D* = 2.788 (2) Å and the distance to the triazine mean plane *d'* = 2.699 (1) Å; hence, F7 is off-centered by 0.700 Å and the incliniation of the *D* vector to the triazine plane is α = 75.5° (*D*, *d'*, and α according to the definitions of Mooibroek *et al.*, 2008). The three shortest distances of F7 to ring atoms are N1 3.012 (2), C1 2.750 (2), N2 2.915 (2) Å. Compared with literature data this is among the shortest F–π-interactions reported so far (Gamez *et al.*, 2007; Mooibroek *et al.*, 2008; Manzano *et al.*, 2008, Quinonero *et al.*, 2010; Lu *et al.*, 2009). For instance, Mooibroek *et al.* (2008) report in their exhaustive CSD date base analysis a mean value of *D* = 3.060±0.184 Å for eight crystal structures with BF_4_ anions and 1,3,5-triazines, and a corresponding value of *D* = 3.076±0.185 Å for 26 cystal structures with PF_6_ anions. The remaining anion–π interactions in (I) are less pronounced but still of interest although they do not concern aromatic rings rather than acetonitrile C≡N groups. Referring to the van der Waals radii sums for F···C = 3.17 Å and F···N = 3.02 Å (Mooibroek *et al.*, 2008), the associations F1···C28 = 3.006 (2) Å and F6···C30 = 3.091 (2) Å suggest anion–π(C≡N) interactions, while F1···C32 = 3.221 (2) Å is slightly above the limit. The corresponding F···N distances are all distinctly larger than the F···C distances and the F···N radii sum.

A packing diagram of the structure is presented in Fig. 3. It gives only the N—H···N hydrogen bonds showing that they link two Fe PNP complexes and two noncoordinating CH_3_CN molecules into a centrosymmetric dimer. The C—H···F and anion–π interactions link all constituents in a three-dimensional supramolecular fashion. π–π stacking between arene rings is lacking in (I).

### Experimental

The title compound, (I), was synthesized by a three-step procedure. 2,6-Diamino-4-ethoxy-1,3,5-triazine: 2,6-Diamino-4-chloro-1,3,5-triazine (3.0 g, 20.6 mmol, Sigma-Aldrich) was suspended in 40 ml of EtOH and treated with KOH (1.27 g, 22.7 mmol) and refluxed for 20 h. The hot solution was then filtered and left in the refrigerator overnight. The supernatant was decanted and the resulting white solid was dried under vacuum. Yield: 2.62 g (82%).

*N*,*N*'-Bis(diphenylphosphino)-2,6-diamino-4-ethoxy-1,3,5-triazine: Triethylamine (2.69 ml, 19.33 mmol) was added to a solution of 2,6-diamino-4-ethoxy-1,3,5-triazine (1.50 g, 9.67 mmol) in toluene (50 ml). The mixture was cooled to 0 °C and PPh_2_Cl (3.47 ml,19.33 mmol) was added dropwise. The reaction was allowed to reach room temperature and refluxed overnight. After that, the solution was filtered and the solvent was removed under vacuum to give a white solid which was dried under vacuum. Yield: 4.61 g (92%).

(I): A solution of *N*,*N*'-bis(diphenylphosphino)-2,6-diamino-4-ethoxy-1,3,5-triazine (0.60 g, 1.15 mmol) and [Fe(H_2_O)_6_](BF_4_)_2_ (0.39 g, 1.15 mmol) in acetonitrile (10 ml) was stirred at room temperature for 4 h. Insoluble materials were then removed by filtration and the volume of the solution was reduced to about 2 ml. The solvent was removed under vacuum and the remaining solid washed twice with Et_2_O. The crude product was purified by flash chromatography (neutral Al_2_O_3_, eluent CH_3_CN). Yield after solvent removal under vacuum: 0.82 g (82%). Orange crystals for X-ray diffraction were obtained by recrystallization from acetonitrile using Et_2_O as the anti-solvent and the vapour diffusion method.

### Refinement

H atoms were located in a difference Fourier map, placed in calculated positions (N—H = 0.88 Å, C—H = 0.95 - 0.98 Å) and thereafter treated as riding. A torsional parameter was refined for each methyl group. *U*_iso_(H) = 1.2*U*_eq_(C,*N*) for CH, CH_2_ and NH groups; *U*_iso_(H) = 1.5*U*_eq_(C) for CH_3_ groups.

### Crystal data

**Table:**

[Fe(C_2_H_3_N)_3_(C_29_H_27_N_5_OP_2_)](BF_4_)_2_·2C_2_H_3_N	*Z* = 2
*M**_r_* = 958.24	*F*(000) = 984
Triclinic, *P*1	*D*_x_ = 1.425 Mg m^−^^3^
Hall symbol: -P 1	Mo *K*α radiation, λ = 0.71073 Å
*a* = 8.8548 (4) Å	Cell parameters from 8213 reflections
*b* = 13.8402 (7) Å	θ = 2.5–30.0°
*c* = 20.1352 (10) Å	µ = 0.49 mm^−^^1^
α = 71.399 (2)°	*T* = 100 K
β = 82.731 (2)°	Block, orange
γ = 72.789 (2)°	0.58 × 0.36 × 0.30 mm
*V* = 2232.6 (2) Å^3^	

### Data collection

**Table:**

Bruker SMART APEX CCD diffractometer	12688 independent reflections
Radiation source: fine-focus sealed tube	11076 reflections with *I* > 2σ(*I*)
graphite	*R*_int_ = 0.017
ω scans	θ_max_ = 30.0°, θ_min_ = 2.5°
Absorption correction: multi-scan (*SADABS*; Bruker, 2003)	*h* = −12→12
*T*_min_ = 0.74, *T*_max_ = 0.86	*k* = −17→19
26262 measured reflections	*l* = −28→28

### Refinement

**Table:**

Refinement on *F*^2^	Primary atom site location: structure-invariant direct methods
Least-squares matrix: full	Secondary atom site location: difference Fourier map
*R*[*F*^2^ > 2σ(*F*^2^)] = 0.038	Hydrogen site location: inferred from neighbouring sites
*wR*(*F*^2^) = 0.102	H-atom parameters constrained
*S* = 1.03	*w* = 1/[σ^2^(*F*_o_^2^) + (0.0577*P*)^2^ + 0.9995*P*] where *P* = (*F*_o_^2^ + 2*F*_c_^2^)/3
12688 reflections	(Δ/σ)_max_ = 0.002
573 parameters	Δρ_max_ = 0.74 e Å^−^^3^
0 restraints	Δρ_min_ = −0.44 e Å^−^^3^

### Special details

**Table:**

Geometry. All e.s.d.'s (except the e.s.d. in the dihedral angle between two l.s. planes) are estimated using the full covariance matrix. The cell e.s.d.'s are taken into account individually in the estimation of e.s.d.'s in distances, angles and torsion angles; correlations between e.s.d.'s in cell parameters are only used when they are defined by crystal symmetry. An approximate (isotropic) treatment of cell e.s.d.'s is used for estimating e.s.d.'s involving l.s. planes.
Refinement. Refinement of *F*^2^ against ALL reflections. The weighted *R*-factor *wR* and goodness of fit *S* are based on *F*^2^, conventional *R*-factors *R* are based on *F*, with *F* set to zero for negative *F*^2^. The threshold expression of *F*^2^ > σ(*F*^2^) is used only for calculating *R*-factors(gt) *etc*. and is not relevant to the choice of reflections for refinement. *R*-factors based on *F*^2^ are statistically about twice as large as those based on *F*, and *R*- factors based on ALL data will be even larger.

### Fractional atomic coordinates and isotropic or equivalent isotropic displacement parameters (Å^2^)

**Table:**

	*x*	*y*	*z*	*U*_iso_*/*U*_eq_	
Fe1	0.54460 (2)	0.690690 (14)	0.254170 (9)	0.01419 (5)	
P1	0.53830 (4)	0.61385 (3)	0.172145 (16)	0.01517 (7)	
P2	0.54736 (4)	0.72777 (3)	0.354940 (17)	0.01594 (7)	
O1	0.37867 (13)	0.30944 (8)	0.44745 (5)	0.0222 (2)	
N1	0.49244 (12)	0.56374 (8)	0.31769 (5)	0.01485 (19)	
N2	0.41916 (13)	0.40539 (9)	0.33384 (6)	0.0170 (2)	
N3	0.44824 (13)	0.45696 (9)	0.43304 (6)	0.0180 (2)	
N4	0.47666 (13)	0.50736 (9)	0.22341 (5)	0.0166 (2)	
H4N	0.4542	0.4641	0.2046	0.020*	
N5	0.50809 (13)	0.61675 (9)	0.41348 (6)	0.0172 (2)	
H5N	0.5047	0.6076	0.4589	0.021*	
N6	0.59524 (13)	0.81386 (9)	0.18835 (6)	0.0181 (2)	
N7	0.76688 (13)	0.62306 (9)	0.26069 (6)	0.0186 (2)	
N8	0.32155 (13)	0.75857 (9)	0.24727 (6)	0.0174 (2)	
C1	0.46287 (14)	0.49058 (10)	0.29390 (6)	0.0156 (2)	
C2	0.41678 (15)	0.39247 (10)	0.40219 (7)	0.0175 (2)	
C3	0.48238 (14)	0.54323 (10)	0.38838 (6)	0.0158 (2)	
C4	0.72769 (15)	0.55979 (11)	0.13392 (7)	0.0190 (2)	
C5	0.79139 (17)	0.63019 (12)	0.07949 (8)	0.0250 (3)	
H5	0.7292	0.7005	0.0594	0.030*	
C6	0.94560 (19)	0.59708 (14)	0.05485 (9)	0.0343 (4)	
H6	0.9893	0.6448	0.0180	0.041*	
C7	1.0360 (2)	0.49401 (16)	0.08419 (10)	0.0397 (4)	
H7	1.1416	0.4714	0.0672	0.048*	
C8	0.9733 (2)	0.42420 (15)	0.13793 (11)	0.0412 (4)	
H8	1.0355	0.3537	0.1575	0.049*	
C9	0.81891 (18)	0.45712 (13)	0.16341 (9)	0.0304 (3)	
H9	0.7763	0.4095	0.2008	0.036*	
C10	0.41181 (15)	0.67008 (10)	0.09752 (7)	0.0177 (2)	
C11	0.32052 (16)	0.77537 (11)	0.08017 (7)	0.0198 (2)	
H11	0.3232	0.8188	0.1080	0.024*	
C12	0.22525 (16)	0.81685 (11)	0.02197 (7)	0.0225 (3)	
H12	0.1615	0.8881	0.0108	0.027*	
C13	0.22351 (17)	0.75411 (12)	−0.01961 (7)	0.0246 (3)	
H13	0.1597	0.7829	−0.0596	0.030*	
C14	0.31498 (18)	0.64906 (12)	−0.00290 (7)	0.0248 (3)	
H14	0.3132	0.6063	−0.0313	0.030*	
C15	0.40884 (17)	0.60697 (11)	0.05546 (7)	0.0217 (3)	
H15	0.4711	0.5354	0.0669	0.026*	
C16	0.72838 (16)	0.72989 (11)	0.38677 (7)	0.0206 (2)	
C17	0.75249 (19)	0.82516 (13)	0.38679 (8)	0.0269 (3)	
H17	0.6729	0.8899	0.3709	0.032*	
C18	0.8950 (2)	0.82454 (16)	0.41049 (9)	0.0363 (4)	
H18	0.9121	0.8891	0.4105	0.044*	
C19	1.0107 (2)	0.73052 (17)	0.43381 (9)	0.0394 (4)	
H19	1.1063	0.7306	0.4504	0.047*	
C20	0.9880 (2)	0.63569 (15)	0.43311 (9)	0.0349 (4)	
H20	1.0687	0.5714	0.4485	0.042*	
C21	0.84664 (18)	0.63495 (13)	0.40975 (8)	0.0262 (3)	
H21	0.8307	0.5701	0.4095	0.031*	
C22	0.39861 (16)	0.84083 (10)	0.37039 (7)	0.0188 (2)	
C23	0.35655 (19)	0.93268 (11)	0.31328 (8)	0.0254 (3)	
H23	0.4041	0.9334	0.2682	0.030*	
C24	0.2454 (2)	1.02256 (12)	0.32279 (9)	0.0301 (3)	
H24	0.2179	1.0848	0.2842	0.036*	
C25	0.17431 (18)	1.02166 (12)	0.38861 (9)	0.0273 (3)	
H25	0.0985	1.0832	0.3950	0.033*	
C26	0.21456 (18)	0.93031 (12)	0.44505 (8)	0.0257 (3)	
H26	0.1652	0.9294	0.4899	0.031*	
C27	0.32680 (16)	0.84016 (11)	0.43620 (7)	0.0213 (2)	
H27	0.3544	0.7782	0.4750	0.026*	
C28	0.62945 (17)	0.88174 (11)	0.14559 (7)	0.0217 (3)	
C29	0.6739 (2)	0.96728 (13)	0.08967 (8)	0.0333 (3)	
H29A	0.7501	0.9373	0.0568	0.050*	
H29B	0.7220	1.0062	0.1100	0.050*	
H29C	0.5795	1.0156	0.0647	0.050*	
C30	0.89967 (17)	0.58461 (12)	0.26125 (8)	0.0247 (3)	
C31	1.07032 (19)	0.53667 (16)	0.26182 (11)	0.0416 (4)	
H31A	1.0920	0.4598	0.2722	0.062*	
H31B	1.1157	0.5524	0.2978	0.062*	
H31C	1.1178	0.5657	0.2158	0.062*	
C32	0.18977 (17)	0.79880 (12)	0.24244 (7)	0.0237 (3)	
C33	0.0217 (2)	0.85205 (18)	0.23351 (11)	0.0480 (5)	
H33A	−0.0404	0.8049	0.2627	0.072*	
H33B	−0.0031	0.8702	0.1842	0.072*	
H33C	−0.0044	0.9168	0.2476	0.072*	
C34	0.33860 (19)	0.23392 (12)	0.42023 (8)	0.0251 (3)	
H34A	0.2883	0.2717	0.3744	0.030*	
H34B	0.2621	0.2010	0.4531	0.030*	
C35	0.4844 (2)	0.14967 (16)	0.41153 (13)	0.0453 (5)	
H35A	0.4561	0.0999	0.3932	0.068*	
H35B	0.5331	0.1116	0.4570	0.068*	
H35C	0.5595	0.1823	0.3786	0.068*	
B1	0.1509 (2)	1.07050 (13)	0.09763 (9)	0.0251 (3)	
F1	0.28453 (14)	0.99849 (9)	0.12992 (8)	0.0545 (4)	
F2	0.07067 (14)	1.13034 (11)	0.14131 (7)	0.0484 (3)	
F3	0.19089 (18)	1.13810 (10)	0.03575 (6)	0.0520 (3)	
F4	0.05329 (13)	1.01714 (8)	0.08354 (6)	0.0381 (2)	
B2	0.9016 (2)	0.31898 (14)	0.36767 (10)	0.0294 (3)	
F5	0.89457 (19)	0.22855 (9)	0.42061 (6)	0.0566 (4)	
F6	0.9380 (2)	0.39051 (10)	0.39354 (7)	0.0579 (4)	
F7	0.76148 (14)	0.36553 (11)	0.33321 (8)	0.0555 (3)	
F8	1.01956 (13)	0.29184 (9)	0.31804 (6)	0.0414 (2)	
N9	0.4500 (2)	0.34626 (12)	0.16629 (8)	0.0379 (3)	
C36	0.4224 (2)	0.28836 (13)	0.14368 (9)	0.0306 (3)	
C37	0.3876 (2)	0.21599 (16)	0.11259 (12)	0.0427 (4)	
H37A	0.4103	0.1447	0.1460	0.064*	
H37B	0.4534	0.2146	0.0698	0.064*	
H37C	0.2757	0.2400	0.1011	0.064*	
N10	0.6755 (2)	0.04815 (13)	0.23086 (9)	0.0414 (4)	
C38	0.7314 (2)	0.10238 (14)	0.24545 (9)	0.0329 (3)	
C39	0.8041 (3)	0.17130 (19)	0.26401 (12)	0.0490 (5)	
H39A	0.7233	0.2363	0.2663	0.073*	
H39B	0.8859	0.1893	0.2285	0.073*	
H39C	0.8521	0.1348	0.3098	0.073*	

### Atomic displacement parameters (Å^2^)

**Table:**

	*U*^11^	*U*^22^	*U*^33^	*U*^12^	*U*^13^	*U*^23^
Fe1	0.01216 (9)	0.01347 (9)	0.01552 (8)	−0.00280 (6)	−0.00222 (6)	−0.00238 (6)
P1	0.01333 (14)	0.01522 (14)	0.01542 (14)	−0.00266 (11)	−0.00135 (10)	−0.00328 (11)
P2	0.01639 (15)	0.01461 (14)	0.01681 (14)	−0.00473 (11)	−0.00263 (11)	−0.00341 (11)
O1	0.0305 (5)	0.0196 (5)	0.0184 (4)	−0.0128 (4)	−0.0006 (4)	−0.0027 (4)
N1	0.0135 (5)	0.0144 (5)	0.0160 (5)	−0.0027 (4)	−0.0017 (3)	−0.0041 (4)
N2	0.0165 (5)	0.0171 (5)	0.0178 (5)	−0.0054 (4)	−0.0026 (4)	−0.0039 (4)
N3	0.0205 (5)	0.0172 (5)	0.0163 (5)	−0.0061 (4)	−0.0021 (4)	−0.0035 (4)
N4	0.0187 (5)	0.0161 (5)	0.0157 (5)	−0.0055 (4)	−0.0015 (4)	−0.0047 (4)
N5	0.0224 (5)	0.0157 (5)	0.0141 (4)	−0.0067 (4)	−0.0021 (4)	−0.0030 (4)
N6	0.0166 (5)	0.0181 (5)	0.0191 (5)	−0.0036 (4)	−0.0031 (4)	−0.0051 (4)
N7	0.0179 (5)	0.0180 (5)	0.0191 (5)	−0.0054 (4)	−0.0035 (4)	−0.0027 (4)
N8	0.0182 (5)	0.0162 (5)	0.0169 (5)	−0.0043 (4)	−0.0014 (4)	−0.0039 (4)
C1	0.0119 (5)	0.0162 (5)	0.0175 (5)	−0.0016 (4)	−0.0033 (4)	−0.0043 (4)
C2	0.0160 (5)	0.0162 (5)	0.0192 (5)	−0.0048 (4)	−0.0022 (4)	−0.0025 (4)
C3	0.0136 (5)	0.0161 (5)	0.0170 (5)	−0.0027 (4)	−0.0027 (4)	−0.0043 (4)
C4	0.0147 (6)	0.0213 (6)	0.0196 (6)	−0.0024 (5)	0.0001 (4)	−0.0066 (5)
C5	0.0201 (6)	0.0246 (7)	0.0257 (6)	−0.0045 (5)	0.0017 (5)	−0.0038 (5)
C6	0.0232 (7)	0.0366 (9)	0.0357 (8)	−0.0076 (6)	0.0085 (6)	−0.0050 (7)
C7	0.0205 (7)	0.0434 (10)	0.0447 (10)	0.0003 (7)	0.0094 (6)	−0.0109 (8)
C8	0.0260 (8)	0.0319 (9)	0.0481 (10)	0.0067 (6)	0.0080 (7)	−0.0049 (7)
C9	0.0222 (7)	0.0241 (7)	0.0340 (8)	0.0006 (5)	0.0045 (6)	−0.0024 (6)
C10	0.0148 (5)	0.0186 (6)	0.0177 (5)	−0.0037 (4)	−0.0021 (4)	−0.0028 (4)
C11	0.0187 (6)	0.0180 (6)	0.0204 (6)	−0.0042 (5)	−0.0017 (4)	−0.0030 (5)
C12	0.0193 (6)	0.0214 (6)	0.0213 (6)	−0.0035 (5)	−0.0032 (5)	−0.0001 (5)
C13	0.0205 (6)	0.0303 (7)	0.0199 (6)	−0.0059 (5)	−0.0053 (5)	−0.0024 (5)
C14	0.0246 (7)	0.0291 (7)	0.0223 (6)	−0.0061 (5)	−0.0043 (5)	−0.0096 (5)
C15	0.0212 (6)	0.0210 (6)	0.0215 (6)	−0.0027 (5)	−0.0034 (5)	−0.0061 (5)
C16	0.0208 (6)	0.0246 (6)	0.0181 (5)	−0.0100 (5)	−0.0034 (4)	−0.0041 (5)
C17	0.0311 (8)	0.0287 (7)	0.0256 (7)	−0.0168 (6)	−0.0019 (5)	−0.0059 (6)
C18	0.0395 (9)	0.0452 (10)	0.0345 (8)	−0.0295 (8)	−0.0038 (7)	−0.0083 (7)
C19	0.0312 (8)	0.0605 (12)	0.0317 (8)	−0.0271 (8)	−0.0087 (6)	−0.0043 (8)
C20	0.0238 (7)	0.0455 (10)	0.0307 (8)	−0.0093 (7)	−0.0107 (6)	−0.0012 (7)
C21	0.0221 (7)	0.0292 (7)	0.0254 (6)	−0.0069 (5)	−0.0069 (5)	−0.0034 (5)
C22	0.0196 (6)	0.0158 (5)	0.0223 (6)	−0.0052 (4)	−0.0033 (4)	−0.0061 (5)
C23	0.0305 (7)	0.0185 (6)	0.0242 (6)	−0.0045 (5)	−0.0016 (5)	−0.0042 (5)
C24	0.0352 (8)	0.0175 (6)	0.0330 (8)	−0.0024 (6)	−0.0050 (6)	−0.0042 (6)
C25	0.0235 (7)	0.0211 (6)	0.0378 (8)	−0.0020 (5)	−0.0040 (6)	−0.0121 (6)
C26	0.0229 (7)	0.0265 (7)	0.0289 (7)	−0.0049 (5)	−0.0002 (5)	−0.0117 (6)
C27	0.0203 (6)	0.0205 (6)	0.0229 (6)	−0.0054 (5)	−0.0024 (5)	−0.0059 (5)
C28	0.0238 (6)	0.0191 (6)	0.0220 (6)	−0.0058 (5)	−0.0006 (5)	−0.0057 (5)
C29	0.0493 (10)	0.0230 (7)	0.0264 (7)	−0.0169 (7)	0.0069 (6)	−0.0024 (6)
C30	0.0189 (6)	0.0245 (7)	0.0287 (7)	−0.0054 (5)	−0.0039 (5)	−0.0043 (5)
C31	0.0157 (7)	0.0446 (10)	0.0570 (11)	−0.0010 (7)	−0.0057 (7)	−0.0098 (9)
C32	0.0206 (6)	0.0239 (6)	0.0205 (6)	−0.0027 (5)	−0.0002 (5)	−0.0016 (5)
C33	0.0188 (8)	0.0557 (12)	0.0434 (10)	0.0061 (7)	0.0010 (7)	0.0049 (9)
C34	0.0328 (7)	0.0229 (7)	0.0247 (6)	−0.0170 (6)	−0.0021 (5)	−0.0047 (5)
C35	0.0432 (10)	0.0356 (9)	0.0685 (13)	−0.0085 (8)	−0.0112 (9)	−0.0292 (9)
B1	0.0253 (8)	0.0208 (7)	0.0277 (7)	−0.0038 (6)	−0.0059 (6)	−0.0054 (6)
F1	0.0397 (6)	0.0273 (5)	0.0937 (10)	0.0018 (5)	−0.0371 (6)	−0.0110 (6)
F2	0.0350 (6)	0.0698 (8)	0.0522 (7)	−0.0098 (6)	0.0023 (5)	−0.0398 (6)
F3	0.0873 (10)	0.0522 (7)	0.0275 (5)	−0.0418 (7)	0.0037 (5)	−0.0080 (5)
F4	0.0372 (5)	0.0315 (5)	0.0483 (6)	−0.0118 (4)	−0.0163 (4)	−0.0078 (4)
B2	0.0302 (9)	0.0228 (8)	0.0311 (8)	−0.0014 (6)	−0.0074 (7)	−0.0052 (6)
F5	0.0906 (10)	0.0270 (5)	0.0376 (6)	−0.0083 (6)	0.0169 (6)	−0.0043 (5)
F6	0.0923 (11)	0.0402 (7)	0.0487 (7)	−0.0204 (7)	−0.0164 (7)	−0.0158 (5)
F7	0.0288 (6)	0.0589 (8)	0.0708 (8)	0.0107 (5)	−0.0183 (5)	−0.0231 (7)
F8	0.0290 (5)	0.0476 (6)	0.0451 (6)	−0.0080 (5)	0.0026 (4)	−0.0139 (5)
N9	0.0545 (10)	0.0293 (7)	0.0345 (7)	−0.0144 (7)	−0.0054 (6)	−0.0115 (6)
C36	0.0304 (8)	0.0252 (7)	0.0371 (8)	−0.0069 (6)	−0.0031 (6)	−0.0106 (6)
C37	0.0330 (9)	0.0388 (10)	0.0683 (13)	−0.0096 (7)	−0.0059 (8)	−0.0308 (9)
N10	0.0441 (9)	0.0342 (8)	0.0459 (9)	−0.0092 (7)	−0.0028 (7)	−0.0128 (7)
C38	0.0322 (8)	0.0284 (8)	0.0324 (8)	−0.0019 (6)	−0.0014 (6)	−0.0071 (6)
C39	0.0472 (12)	0.0539 (12)	0.0558 (12)	−0.0206 (10)	0.0016 (9)	−0.0248 (10)

### Geometric parameters (Å, °)

**Table:**

Fe1—N1	1.9579 (11)	C18—H18	0.9500
Fe1—N6	1.9298 (11)	C19—C20	1.390 (3)
Fe1—N7	1.9142 (12)	C19—H19	0.9500
Fe1—N8	1.9211 (11)	C20—C21	1.395 (2)
Fe1—P1	2.2452 (4)	C20—H20	0.9500
Fe1—P2	2.2506 (4)	C21—H21	0.9500
P1—N4	1.7025 (11)	C22—C27	1.3938 (19)
P1—C4	1.8063 (13)	C22—C23	1.4046 (19)
P1—C10	1.8116 (13)	C23—C24	1.390 (2)
P2—N5	1.7113 (11)	C23—H23	0.9500
P2—C22	1.8075 (14)	C24—C25	1.391 (2)
P2—C16	1.8113 (14)	C24—H24	0.9500
O1—C2	1.3222 (15)	C25—C26	1.391 (2)
O1—C34	1.4673 (17)	C25—H25	0.9500
N1—C1	1.3495 (16)	C26—C27	1.393 (2)
N1—C3	1.3564 (16)	C26—H26	0.9500
N2—C2	1.3284 (16)	C27—H27	0.9500
N2—C1	1.3315 (16)	C28—C29	1.4620 (19)
N3—C3	1.3362 (16)	C29—H29A	0.9800
N3—C2	1.3434 (17)	C29—H29B	0.9800
N4—C1	1.3587 (16)	C29—H29C	0.9800
N4—H4N	0.8800	C30—C31	1.459 (2)
N5—C3	1.3553 (16)	C31—H31A	0.9800
N5—H5N	0.8800	C31—H31B	0.9800
N6—C28	1.1411 (18)	C31—H31C	0.9800
N7—C30	1.1380 (19)	C32—C33	1.457 (2)
N8—C32	1.1361 (19)	C33—H33A	0.9800
C4—C9	1.3885 (19)	C33—H33B	0.9800
C4—C5	1.3987 (19)	C33—H33C	0.9800
C5—C6	1.387 (2)	C34—C35	1.497 (3)
C5—H5	0.9500	C34—H34A	0.9900
C6—C7	1.390 (2)	C34—H34B	0.9900
C6—H6	0.9500	C35—H35A	0.9800
C7—C8	1.382 (3)	C35—H35B	0.9800
C7—H7	0.9500	C35—H35C	0.9800
C8—C9	1.394 (2)	B1—F3	1.376 (2)
C8—H8	0.9500	B1—F1	1.378 (2)
C9—H9	0.9500	B1—F2	1.387 (2)
C10—C11	1.3960 (18)	B1—F4	1.395 (2)
C10—C15	1.4042 (19)	B2—F5	1.373 (2)
C11—C12	1.3961 (18)	B2—F7	1.377 (2)
C11—H11	0.9500	B2—F6	1.384 (2)
C12—C13	1.389 (2)	B2—F8	1.405 (2)
C12—H12	0.9500	N9—C36	1.134 (2)
C13—C14	1.394 (2)	C36—C37	1.457 (2)
C13—H13	0.9500	C37—H37A	0.9800
C14—C15	1.3911 (19)	C37—H37B	0.9800
C14—H14	0.9500	C37—H37C	0.9800
C15—H15	0.9500	N10—C38	1.137 (2)
C16—C17	1.397 (2)	C38—C39	1.454 (3)
C16—C21	1.399 (2)	C39—H39A	0.9800
C17—C18	1.401 (2)	C39—H39B	0.9800
C17—H17	0.9500	C39—H39C	0.9800
C18—C19	1.381 (3)		
			
N7—Fe1—N8	179.78 (5)	C16—C17—H17	120.3
N7—Fe1—N6	88.04 (5)	C18—C17—H17	120.3
N8—Fe1—N6	91.87 (5)	C19—C18—C17	120.32 (16)
N7—Fe1—N1	92.09 (5)	C19—C18—H18	119.8
N8—Fe1—N1	87.99 (4)	C17—C18—H18	119.8
N6—Fe1—N1	177.64 (4)	C18—C19—C20	120.39 (15)
N7—Fe1—P1	88.65 (4)	C18—C19—H19	119.8
N8—Fe1—P1	91.16 (3)	C20—C19—H19	119.8
N6—Fe1—P1	94.65 (3)	C19—C20—C21	119.99 (16)
N1—Fe1—P1	83.00 (3)	C19—C20—H20	120.0
N7—Fe1—P2	89.37 (4)	C21—C20—H20	120.0
N8—Fe1—P2	90.84 (3)	C20—C21—C16	119.83 (15)
N6—Fe1—P2	99.61 (3)	C20—C21—H21	120.1
N1—Fe1—P2	82.74 (3)	C16—C21—H21	120.1
P1—Fe1—P2	165.523 (14)	C27—C22—C23	119.57 (13)
N4—P1—C4	105.65 (6)	C27—C22—P2	122.65 (10)
N4—P1—C10	105.10 (6)	C23—C22—P2	117.78 (11)
C4—P1—C10	102.57 (6)	C24—C23—C22	119.99 (14)
N4—P1—Fe1	99.37 (4)	C24—C23—H23	120.0
C4—P1—Fe1	115.85 (5)	C22—C23—H23	120.0
C10—P1—Fe1	126.17 (5)	C23—C24—C25	120.26 (14)
N5—P2—C22	107.23 (6)	C23—C24—H24	119.9
N5—P2—C16	102.50 (6)	C25—C24—H24	119.9
C22—P2—C16	105.16 (6)	C24—C25—C26	119.79 (14)
N5—P2—Fe1	99.62 (4)	C24—C25—H25	120.1
C22—P2—Fe1	118.48 (4)	C26—C25—H25	120.1
C16—P2—Fe1	121.60 (5)	C25—C26—C27	120.39 (14)
C2—O1—C34	118.51 (11)	C25—C26—H26	119.8
C1—N1—C3	115.24 (11)	C27—C26—H26	119.8
C1—N1—Fe1	122.07 (8)	C26—C27—C22	120.00 (13)
C3—N1—Fe1	122.69 (9)	C26—C27—H27	120.0
C2—N2—C1	114.05 (11)	C22—C27—H27	120.0
C3—N3—C2	114.42 (11)	N6—C28—C29	178.68 (15)
C1—N4—P1	118.59 (9)	C28—C29—H29A	109.5
C1—N4—H4N	120.7	C28—C29—H29B	109.5
P1—N4—H4N	120.7	H29A—C29—H29B	109.5
C3—N5—P2	118.55 (9)	C28—C29—H29C	109.5
C3—N5—H5N	120.7	H29A—C29—H29C	109.5
P2—N5—H5N	120.7	H29B—C29—H29C	109.5
C28—N6—Fe1	174.22 (12)	N7—C30—C31	179.24 (17)
C30—N7—Fe1	176.80 (12)	C30—C31—H31A	109.5
C32—N8—Fe1	179.27 (11)	C30—C31—H31B	109.5
N2—C1—N1	125.14 (11)	H31A—C31—H31B	109.5
N2—C1—N4	118.25 (11)	C30—C31—H31C	109.5
N1—C1—N4	116.59 (11)	H31A—C31—H31C	109.5
O1—C2—N2	119.85 (12)	H31B—C31—H31C	109.5
O1—C2—N3	113.21 (11)	N8—C32—C33	177.90 (16)
N2—C2—N3	126.93 (12)	C32—C33—H33A	109.5
N3—C3—N5	119.61 (11)	C32—C33—H33B	109.5
N3—C3—N1	124.04 (11)	H33A—C33—H33B	109.5
N5—C3—N1	116.35 (11)	C32—C33—H33C	109.5
C9—C4—C5	120.05 (13)	H33A—C33—H33C	109.5
C9—C4—P1	122.06 (11)	H33B—C33—H33C	109.5
C5—C4—P1	117.17 (10)	O1—C34—C35	110.26 (13)
C6—C5—C4	119.86 (14)	O1—C34—H34A	109.6
C6—C5—H5	120.1	C35—C34—H34A	109.6
C4—C5—H5	120.1	O1—C34—H34B	109.6
C5—C6—C7	119.87 (15)	C35—C34—H34B	109.6
C5—C6—H6	120.1	H34A—C34—H34B	108.1
C7—C6—H6	120.1	C34—C35—H35A	109.5
C8—C7—C6	120.41 (15)	C34—C35—H35B	109.5
C8—C7—H7	119.8	H35A—C35—H35B	109.5
C6—C7—H7	119.8	C34—C35—H35C	109.5
C7—C8—C9	120.08 (16)	H35A—C35—H35C	109.5
C7—C8—H8	120.0	H35B—C35—H35C	109.5
C9—C8—H8	120.0	F3—B1—F1	110.60 (15)
C4—C9—C8	119.74 (15)	F3—B1—F2	108.26 (14)
C4—C9—H9	120.1	F1—B1—F2	109.22 (14)
C8—C9—H9	120.1	F3—B1—F4	109.10 (13)
C11—C10—C15	119.56 (12)	F1—B1—F4	109.80 (13)
C11—C10—P1	121.28 (10)	F2—B1—F4	109.85 (14)
C15—C10—P1	119.15 (10)	F5—B2—F7	111.28 (17)
C10—C11—C12	120.02 (13)	F5—B2—F6	110.76 (15)
C10—C11—H11	120.0	F7—B2—F6	109.86 (15)
C12—C11—H11	120.0	F5—B2—F8	108.77 (14)
C13—C12—C11	120.11 (13)	F7—B2—F8	106.97 (14)
C13—C12—H12	119.9	F6—B2—F8	109.10 (16)
C11—C12—H12	119.9	N9—C36—C37	178.3 (2)
C12—C13—C14	120.25 (12)	C36—C37—H37A	109.5
C12—C13—H13	119.9	C36—C37—H37B	109.5
C14—C13—H13	119.9	H37A—C37—H37B	109.5
C15—C14—C13	119.90 (13)	C36—C37—H37C	109.5
C15—C14—H14	120.1	H37A—C37—H37C	109.5
C13—C14—H14	120.1	H37B—C37—H37C	109.5
C14—C15—C10	120.16 (13)	N10—C38—C39	179.6 (2)
C14—C15—H15	119.9	C38—C39—H39A	109.5
C10—C15—H15	119.9	C38—C39—H39B	109.5
C17—C16—C21	119.97 (13)	H39A—C39—H39B	109.5
C17—C16—P2	120.80 (11)	C38—C39—H39C	109.5
C21—C16—P2	119.21 (11)	H39A—C39—H39C	109.5
C16—C17—C18	119.50 (16)	H39B—C39—H39C	109.5
			
N7—Fe1—P1—N4	97.16 (5)	C1—N1—C3—N3	0.95 (18)
N8—Fe1—P1—N4	−82.94 (5)	Fe1—N1—C3—N3	−179.47 (9)
N6—Fe1—P1—N4	−174.91 (5)	C1—N1—C3—N5	−178.57 (11)
N1—Fe1—P1—N4	4.89 (5)	Fe1—N1—C3—N5	1.02 (16)
P2—Fe1—P1—N4	14.95 (7)	N4—P1—C4—C9	−21.11 (14)
N7—Fe1—P1—C4	−15.41 (6)	C10—P1—C4—C9	−130.97 (13)
N8—Fe1—P1—C4	164.49 (6)	Fe1—P1—C4—C9	87.77 (13)
N6—Fe1—P1—C4	72.52 (6)	N4—P1—C4—C5	168.62 (11)
N1—Fe1—P1—C4	−107.68 (6)	C10—P1—C4—C5	58.76 (12)
P2—Fe1—P1—C4	−97.62 (7)	Fe1—P1—C4—C5	−82.50 (11)
N7—Fe1—P1—C10	−146.23 (6)	C9—C4—C5—C6	0.3 (2)
N8—Fe1—P1—C10	33.66 (6)	P1—C4—C5—C6	170.76 (13)
N6—Fe1—P1—C10	−58.31 (6)	C4—C5—C6—C7	0.2 (3)
N1—Fe1—P1—C10	121.50 (6)	C5—C6—C7—C8	−0.1 (3)
P2—Fe1—P1—C10	131.56 (7)	C6—C7—C8—C9	−0.5 (3)
N7—Fe1—P2—N5	−90.44 (5)	C5—C4—C9—C8	−0.8 (3)
N8—Fe1—P2—N5	89.62 (5)	P1—C4—C9—C8	−170.83 (15)
N6—Fe1—P2—N5	−178.34 (5)	C7—C8—C9—C4	0.9 (3)
N1—Fe1—P2—N5	1.75 (5)	N4—P1—C10—C11	122.93 (11)
P1—Fe1—P2—N5	−8.32 (7)	C4—P1—C10—C11	−126.80 (11)
N7—Fe1—P2—C22	153.87 (6)	Fe1—P1—C10—C11	8.96 (13)
N8—Fe1—P2—C22	−26.08 (6)	N4—P1—C10—C15	−58.64 (12)
N6—Fe1—P2—C22	65.96 (6)	C4—P1—C10—C15	51.64 (12)
N1—Fe1—P2—C22	−113.95 (6)	Fe1—P1—C10—C15	−172.61 (9)
P1—Fe1—P2—C22	−124.01 (7)	C15—C10—C11—C12	1.0 (2)
N7—Fe1—P2—C16	20.79 (6)	P1—C10—C11—C12	179.40 (10)
N8—Fe1—P2—C16	−159.16 (6)	C10—C11—C12—C13	−1.3 (2)
N6—Fe1—P2—C16	−67.12 (6)	C11—C12—C13—C14	0.9 (2)
N1—Fe1—P2—C16	112.97 (6)	C12—C13—C14—C15	−0.2 (2)
P1—Fe1—P2—C16	102.91 (8)	C13—C14—C15—C10	−0.1 (2)
N7—Fe1—N1—C1	−93.16 (10)	C11—C10—C15—C14	−0.3 (2)
N8—Fe1—N1—C1	86.64 (10)	P1—C10—C15—C14	−178.74 (11)
P1—Fe1—N1—C1	−4.78 (9)	N5—P2—C16—C17	−140.20 (12)
P2—Fe1—N1—C1	177.74 (10)	C22—P2—C16—C17	−28.22 (13)
N7—Fe1—N1—C3	87.28 (10)	Fe1—P2—C16—C17	110.08 (11)
N8—Fe1—N1—C3	−92.93 (10)	N5—P2—C16—C21	41.53 (13)
P1—Fe1—N1—C3	175.66 (10)	C22—P2—C16—C21	153.51 (11)
P2—Fe1—N1—C3	−1.82 (9)	Fe1—P2—C16—C21	−68.19 (12)
C4—P1—N4—C1	114.59 (10)	C21—C16—C17—C18	−0.5 (2)
C10—P1—N4—C1	−137.37 (10)	P2—C16—C17—C18	−178.79 (12)
Fe1—P1—N4—C1	−5.76 (10)	C16—C17—C18—C19	−0.1 (2)
C22—P2—N5—C3	122.01 (10)	C17—C18—C19—C20	0.9 (3)
C16—P2—N5—C3	−127.55 (10)	C18—C19—C20—C21	−1.0 (3)
Fe1—P2—N5—C3	−1.97 (10)	C19—C20—C21—C16	0.3 (2)
C2—N2—C1—N1	−4.55 (18)	C17—C16—C21—C20	0.5 (2)
C2—N2—C1—N4	176.68 (11)	P2—C16—C21—C20	178.74 (12)
C3—N1—C1—N2	3.11 (18)	N5—P2—C22—C27	29.94 (13)
Fe1—N1—C1—N2	−176.49 (9)	C16—P2—C22—C27	−78.65 (12)
C3—N1—C1—N4	−178.10 (10)	Fe1—P2—C22—C27	141.48 (10)
Fe1—N1—C1—N4	2.30 (15)	N5—P2—C22—C23	−150.77 (11)
P1—N4—C1—N2	−178.03 (9)	C16—P2—C22—C23	100.64 (12)
P1—N4—C1—N1	3.10 (15)	Fe1—P2—C22—C23	−39.23 (13)
C34—O1—C2—N2	−0.48 (18)	C27—C22—C23—C24	0.8 (2)
C34—O1—C2—N3	178.84 (12)	P2—C22—C23—C24	−178.52 (12)
C1—N2—C2—O1	−178.51 (11)	C22—C23—C24—C25	−0.6 (2)
C1—N2—C2—N3	2.27 (19)	C23—C24—C25—C26	−0.1 (2)
C3—N3—C2—O1	−178.05 (11)	C24—C25—C26—C27	0.7 (2)
C3—N3—C2—N2	1.2 (2)	C25—C26—C27—C22	−0.5 (2)
C2—N3—C3—N5	176.61 (12)	C23—C22—C27—C26	−0.2 (2)
C2—N3—C3—N1	−2.89 (18)	P2—C22—C27—C26	179.05 (11)
P2—N5—C3—N3	−178.61 (9)	C2—O1—C34—C35	89.18 (17)
P2—N5—C3—N1	0.93 (16)		

### Hydrogen-bond geometry (Å, °)

**Table:**

*D*—H···*A*	*D*—H	H···*A*	*D*···*A*	*D*—H···*A*
N4—H4N···N9	0.88	2.03	2.892 (2)	168
N5—H5N···N3^i^	0.88	2.12	2.966 (2)	162
C9—H9···F7	0.95	2.53	3.276 (2)	135
C12—H12···F4	0.95	2.56	3.280 (2)	133
C13—H13···F2^ii^	0.95	2.54	3.464 (2)	164
C21—H21···F6	0.95	2.49	3.350 (2)	150
C25—H25···F5^iii^	0.95	2.42	3.365 (2)	174
C26—H26···F5^i^	0.95	2.50	3.144 (2)	125
C29—H29A···F3^iv^	0.98	2.35	3.239 (2)	151
C31—H31A···F8	0.98	2.46	3.360 (2)	153
C33—H33B···F4	0.98	2.49	3.191 (2)	128
C39—H39B···F2^v^	0.98	2.41	3.259 (3)	144

Symmetry codes: (i) −*x*+1, −*y*+1, −*z*+1; (ii) −*x*, −*y*+2, −*z*; (iii) *x*−1, *y*+1, *z*; (iv) −*x*+1, −*y*+2, −*z*; (v) *x*+1, *y*−1, *z*.
